# Femtosecond Core-Level Charge Transfer

**DOI:** 10.1021/acs.jpclett.5c01485

**Published:** 2025-08-21

**Authors:** Simon P. Neville, Martha Yaghoubi Jouybari, Michael S. Schuurman

**Affiliations:** † National Research Council Canada, 100 Sussex Drive, Ottawa, Ontario K1A 0R6, Canada; ‡ Department of Chemistry and Biomolecular Sciences, University of Ottawa, 10 Marie Curie, Ottawa, Ontario K1N 6N5, Canada

## Abstract

We predict that ultrafast, few-femtosecond (fs) core-level charge transfer may occur following X-ray excitation, driven by nonadiabatic dynamics. Using the example of ethylene excited to its 1sπ* manifold, we predict that a transfer of core-electron density from one side of the molecule to the other occurs within 5 fs following broadband X-ray excitation, resulting in core-hole localization. These dynamics are predicted to occur within the Auger decay window, thus, in principle, rendering them observable.

## Graphical abstract

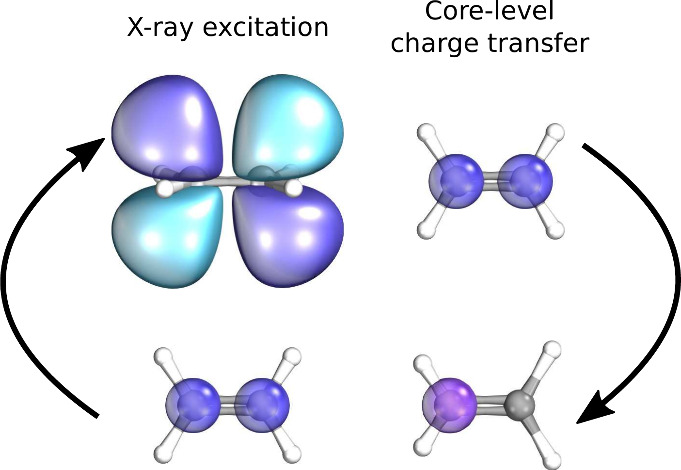

The transfer of charge through a molecular system is of fundamental importance in a range of physical, chemical, and biological processes, including charge injection in solar cells and photosynthesis. Broadly speaking, there exist two principal mechanisms for the redistribution of charge through a molecular system: charge migration and charge transfer. In charge migration,^1–11^ the interaction with a broadband laser pulse creates a coherent superposition of multiple electronic states, leading to coherent electronic dynamics and a redistribution of electron density over the molecule. These dynamics are purely electronic in nature, insofar as they would still occur even if the nuclei were rendered static. Charge transfer, on the other hand, is driven by nuclear dynamics, with there existing no initial electronic coherence created by the pump laser pulse. Instead, a single electronic state is initially populated and the redistribution of electronic density is occurs due to nonadiabatic coupled nuclear-electronic dynamics. Charge migration, being purely electronic in nature, can occur on a few-femtosecond (fs) time scale. Charge transfer, arising from comparatively slower nuclear motion, in general occurs on a longer time scale. There are some exceptions to this, notably the prediction of few-fs double valence-hole transfer in glycine following core-ionization and subsequent Auger decay.^12^ We here demonstrate another exception, showing that ultrafast (few-fs) nuclear-motion-driven core-level charge transfer may occur following core-excitation by an ultrafast X-ray laser pulse.

We consider X-ray absorption in molecules containing two or more equivalent core atomic orbitals (AOs). This covers broad classes of organic and inorganic molecules of fundamental importance, e.g., the polyenes, acenes, and many transition metal complexes. For *N* equivalent core AOs, core-excitation will give rise to sets of *N* near-degenerate core-excited states, each corresponding to excitation to the same final virtual orbital. From the pioneering work of the Köppel, Schirmer, Cederbaum and co-workers,^13,14^ it is known that strong vibronic coupling exists within such manifolds of nearly degenerate core-excited states, and that this can result in core-hole localization in the corresponding vibronic states. This raises the appealing prospect of broadband X-ray absorption giving rise to core-hole redistribution driven by nuclear motion. That is, core-level charge transfer. We here show that this is indeed the case, and that core-level charge transfer can occur on an ultrafast (few-fs) time scale. Importantly, we demonstrate that these core-hole dynamics may occur within the Auger decay window and are, therefore, observable in principle.

As a representative example, we consider 1*s* → π* excitation in ethylene. At the *D*_2*h*_ ground state minimum energy geometry there exist two equivalent C 1*s* AOs. The symmetric and antisymmetric linear combinations of these gives rise to two near-degenerate C 1*s* molecular orbitals (MOs) of *a*_*g*_ and *b*_3*u*_ symmetry. Excitation from these core-level orbitals to the π* orbital gives rise to the nearly degenerate *B*_1*u*_(1sπ*) and *B*_2*g*_(1sπ*) states. For reference, the dominant natural transition orbitals (NTOs) for transition to these states are shown in the first two columns of Figure 1. The *B*_1*u*_(1sπ*) state is optically bright, while the *B*_2*g*_(1sπ*) state is optically dark. However, these two states are strongly vibronically coupled by the *b*_3*u*_ asymmetric in-plane C–H stretching mode,^13,14^ leading to vibronic states of mixed *B*_1*u*_(1sπ*) and *B*_2*g*_(1sπ*) character. As we shall demonstrate, excitation to the former electronic state using a broadband X-ray pulse leads to the ultrafast transfer of core–electron density from one side of the molecule to the other, resulting in core-hole localization. This is driven by nuclear motion and is a result of the breakdown of the Born–Oppenheimer approximation and, as such, corresponds to a charge transfer process. We note that core-hole localization in ethylene has previously been studied,^13,14^ however, these studies did not consider the time scale of the core-hole localization.

**Figure 1:**
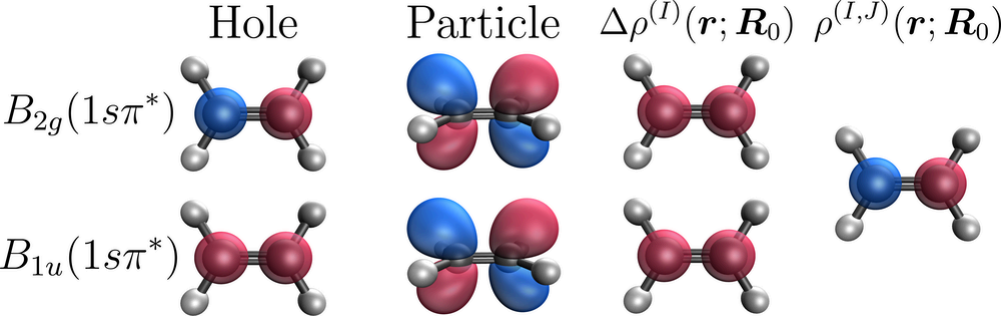
Columns 1 and 2: diabatic hole and particle NTOs computed for the *B*_1*u*_(1sπ*) and *B*_2*g*_(1sπ*) states. Column 3: diabatic one-electron difference densities Δρ^(*I*)^(***r***; ***R***_0_) between the *B*_1*u*_(1sπ*) and *B*_2*g*_(1sπ*) states and the ground state. Column 4: diabatic one-electron transition density ρ^(*I*,*J*)^(***r***; ***R***_0_) between the *B*_1*u*_(1sπ*) and *B*_2*g*_(1sπ*) states. All quantities were calculated at the *D*_2*h*_ ground state minimum energy geometry at the DFT/MRCI(2) level of theory with the def2-TZVP basis.

We begin with the definition of some notation. Let ***r*** and ***R*** denote the vectors of electronic and nuclear degrees of freedom, respectively, and Ψ­(***r***, ***R***, *t*) the time-dependent vibronic wave function. Our starting point is the Born-Huang expansion of Ψ­(***r***, ***R***, *t*) in terms of a set of electronic wave functions {ψ_*I*_(***r***; ***R***)}

$$\Psi \left(r,R,t\right)=\underset{I}{\sum }\chi_{I}\left(R,t\right)\psi_{I}\left(r;R\right)$$ 1

where the χ_*I*_(***R***, *t*) are the nuclear wave functions. In the following, we shall assume a *diabatic* representation as the conservation of the characters of the diabatic electronic wave functions with nuclear geometry allows for a simpler interpretation. For the case considered here, the expansion in eq 1 shall be limited to the *B*_1*u*_(1sπ*) and *B*_2*g*_(1sπ*) states. The time-evolution of the nuclear wave functions encodes any core-level dynamics that may be initiated by X-ray excitation. The question, then, is how to quantify and, ideally, visualize these dynamics. To this end, we note that the Born-Huang wave function *ansatz* allows us to define a *vibronic* one-electron reduced density (1-VRD)

$$\rho \left(r_{1},R,t\right)=n_{el}\int dr_{2}\cdot \cdot \cdot r_{n_{el}}\Psi^{*}\left(r,R,t\right)\Psi \left(r,R,t\right)$$ 2

where *n*_*el*_ is the number of electrons. As the electrons are indistinguishable, we shall subsequently use **r** to denote the coordinates of a single electron instead of ***r***_1_. The 1-VRD contains information about the coupled electron–nuclear dynamics, from which the vibronic core-level dynamics may be extracted.

Inserting the Born-Huang expansion of the wave function into eq 2, we obtain the following expression for the 1-VRD:

$$\rho \left(r,R,t\right)=\underset{I,J}{\sum }\chi_{I}^{*}\left(R,t\right)\chi_{J}\left(R,t\right)\rho^{\left(I,J\right)}\left(r;R\right)$$ 3

where the ρ^(*I*,*J*)^(**r**; ***R***) are the standard electronic one-electron reduced (transition) densities:

$$\rho^{\left(I,J\right)}\left(r;R\right)=n_{el}\int dr_{2}\cdot \cdot \cdot dr_{n_{el}}\psi_{I}^{*}\left(r;R\right)\psi_{J}\left(r;R\right)$$ 4

We next introduce the concept of a “vibronic core-hole”. An intuitive definition is as follows. The difference between 1-VRD ρ­(**r**, ***R***, *t*) and the diabatic ground state one-electron reduced density, ρ_0_(**r**; ***R***), is first computed, yielding the vibronic one-electron difference density Δρ­(**r**, ***R***, *t*):

$$\Delta \rho \left(r,R,t\right)=\rho \left(r,R,t\right)-\alpha \left(R,t\right)\rho_{0}\left(r;R\right)$$ 5

$$\alpha \left(R,t\right)=\frac{1}{n_{el}}\int dr\rho \left(r,R,t\right)$$ 6

where the scaling factor α­(***R***, *t*) ensures that Δρ­(**r**, ***R***, *t*) integrates to zero, as should be the case for an electron-number conserving excitation process. The “core-hole” is then identified with the vibronic core-hole density, Δρ^(*core*)^(**r**, ***R***, *t*), defined by

$$\Delta \rho^{\left(core\right)}\left(r,R,t\right)={\mathrm{P̂}}_{1s}\left(r;R\right)\Delta \rho \left(r,R,t\right)$$ 7

where *P̂*_1*s*_(**r**; ***R***) is the projector onto the subspace spanned by the 1*s* core-level electrons. Inserting the eq 3 into eq 7, and introducing the one-electron difference density Δρ^(*I*)^(**r**; ***R***)­

$$\Delta \rho^{\left(I\right)}\left(r;R\right)=\rho^{\left(I,I\right)}\left(r;R\right)-\rho_{0}\left(r;R\right)$$ 8

we obtain

$$\Delta \rho^{\left(core\right)}\left(r,R,t\right)=\Delta \rho_{pop}^{\left(core\right)}\left(r,R,t\right)+\Delta \rho_{coh}^{\left(core\right)}\left(r,R,t\right)$$ 9

with

$$\Delta \rho_{pop}^{\left(core\right)}\left(r,R,t\right)={\mathrm{P̂}}_{1s}\left(r;R\right)\underset{I}{\sum }|\chi_{I}\left(R,t\right){|}^{2}\Delta \rho^{\left(I\right)}\left(r;R\right)$$ 10

$$\Delta \rho_{coh}^{\left(core\right)}\left(r,R,t\right)={\mathrm{P̂}}_{1s}\left(r;R\right)\underset{I\neq J}{\sum }\chi_{I}^{*}\left(R,t\right)\chi_{J}\left(R,t\right)\rho^{\left(I,J\right)}\left(r;R\right)$$ 11

Thus, the vibronic core-hole density can be decomposed into two contributions: (i) a “population” term, Δρ_*pop*_^(*core*)^(**r**, ***R***, *t*), involving the one-electron difference densities of the core-excited states involved weighted by the corresponding nuclear probability densities and (ii) a “coherence” term, Δρ_*coh*_^(*core*)^(**r**, ***R***, *t*), involving the one-electron transition densities between the core-excited states weighted by the “spatially resolved” electronic coherence χ_I_^*^(***R***, *t*)­χ_*J*_(***R***, *t*). We note that, following Arribas and Maitra,^15^ the term “spatially resolved coherence” is used to distinguish these products from the off-diagonal elements of the electronic reduced density matrix, the so-called “spatially averaged coherences”,^15^ which are also commonly simply termed “electronic coherences” in the literature.

At this point, it is instructive to consider the specific forms of the one-electron difference and transition densities for the *B*_1*u*_(1sπ*) and *B*_2*g*_(1sπ*) states following projection onto the core-orbital subspace. These are shown in Figure 1 as computed at the *D*_2*h*_ ground state geometry, denoted here by ***R***_0_. The third column shows the one-electron difference densities, Δρ^(*I*)^(***r***; ***R***), between the core-excited states and the ground state, while the fourth column shows the one-electron transition density, ρ^(*I*,*J*)^(***r***; ***R***), between the core-excited states. The one-electron difference densities are essentially identical. Since the difference densities contribute to the vibronic core-hole density with positive weights, it is thus clear that the population contribution, Δρ_*pop*_^(*core*)^(**r**, ***R***, *t*), alone will always correspond to a delocalized core-hole. That is, a core-hole that is symmetrically distributed over the two carbon atoms. However, the transfer of core–electron density from one side of the molecule to the other may occur if the coherence contribution, Δρ_*coh*_^(*core*)^(**r**, ***R***, *t*), is non-negligible. This can be seen from the form of the one-electron transition density ρ^(*I*,*J*)^(***r***; ***R***), which contains both positive and negative regions. When added to the population contribution this can lead to the depletion of electron density in the region surrounding one of the two equivalent carbon atoms. That is core-level charge transfer may be driven, leading to core-hole localization. As indicated by eq 11, a requirement for core-hole localization to occur is that the nuclear wave functions of the different electronic states exhibit a high degree of overlap. Whether or not this will happen is contingent on the coupled nuclear-electronic dynamics and, in particular, on whether the two nuclear wave functions overlap significantly during its course.

To simulate the coupled nuclear-electronic dynamics, we adopt a previously demonstrated strategy for simulating short-time dynamics using two effective modes: the gradient difference and nonadiabatic coupling directions, denoted *x* and *y*, respectively.^16^ Due to the fact that (i) the *B*_1*u*_(1sπ*) and *B*_2*g*_(1sπ*) states are quasi-degenerate at the ground state geometry, and (ii) the core-excited states have very short (sub-10 fs) lifetimes due to Auger decay, it is reasonable to expect the nuclear dynamics to be effectively confined to this two-dimensional subspace, which would correspond to a conical intersection branching space if the two states were truly degenerate. Numerically exact wave packet propagations were performed using a vibronic coupling Hamiltonian.^17,18^ The model Hamiltonian was parametrized to the results of QD-DFT/MRCI(2)^19,20^ calculations using the direct fitting procedure detailed in ref 21. All calculations were performed within the core–valence separation (CVS) approximation^22–24^ using the newly introduced CVS-QE12 Hamiltonian.^25^ To confirm the accuracy of the QD-DFT/MRCI(2) calculations, the Franck–Condon vertical core-excitation energies of the *B*_1*u*_(1sπ*) and *B*_2*g*_(1sπ*) states were computed and compared to benchmark-level CCSDT/aug-cc-pCVTZ results, with the two agreeing to within 0.5 eV. The initial wave packet, chosen as the vertical excitation of the ground vibronic state to the optically bright *B*_1*u*_(1sπ*) state, was selected to emulate broadband excitation with a few-fs X-ray laser pulse. The details of the model Hamiltonian, as well as the validity of the effective mode and vertical excitation approximations are demonstrated in the Supporting Information.

We first note that the gradient difference mode *x* is dominated by *a*_*g*_ symmetric C–C stretching, while the nonadiabatic coupling mode *y* corresponds to almost entirely to *b*_3*u*_ asymmetric C–H stretching, in agreement with previous work.^13,14^ Next, we consider the diabatic state populations following excitation to the *B*_1*u*_(1sπ*) state, which are shown in Figure 2. Solid lines are the results of a two-mode calculation, including both *x* and *y*, while the dots are the result of a one-mode calculation including *y* only. We first note that the gradient difference mode *x* has essentially zero effect on the population dynamics, a consequence of a small difference in the *B*_1*u*_(1sπ*) and *B*_2*g*_(1sπ*) potential gradients. That is, the two diabatic potentials remain quasi-degenerate along *x*. Thus, as far as the population contribution to the vibronic core-hole density, Δρ_*pop*_^(*core*)^(**r**, ***R***, *t*), goes, this mode may be safely neglected. Furthermore, as the gradient difference is the principle mechanism for decoherence, this mode will also have little effect on the coherence contribution, Δρ_*coh*_^(*core*)^(**r**, ***R***, *t*). As such, to an excellent approximation, the core-hole dynamics can be modeled using a simple one-mode model including only the nonadiabatic coupling direction *y*, corresponding to asymmetric C–H stretching.

**Figure 2:**
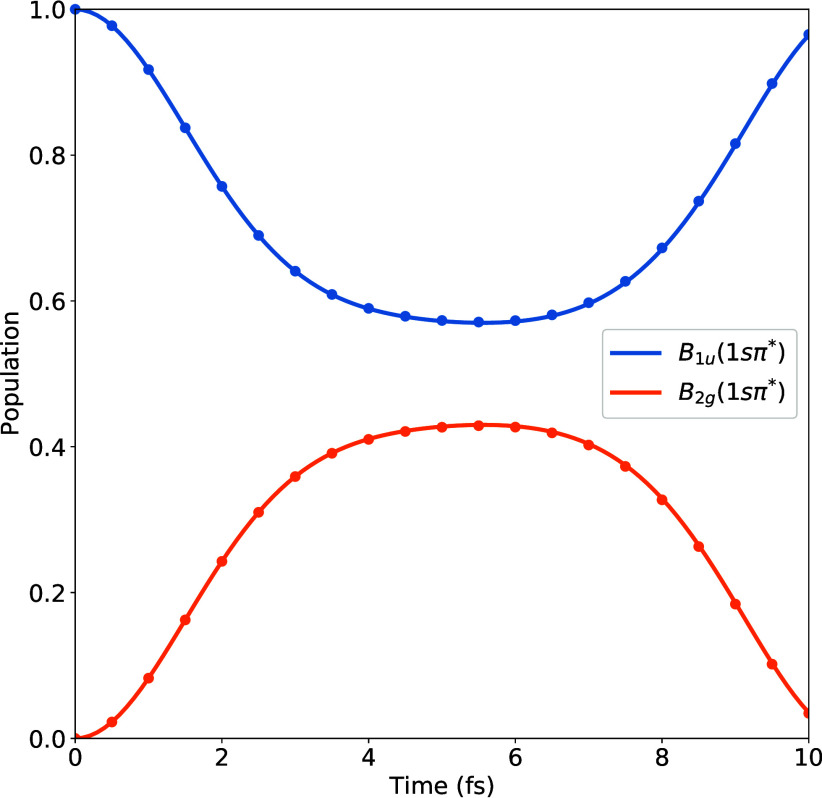
Diabatic state populations following excitation to the *B*_1*u*_(1sπ*) state. Solid lines: results of a wave packet propagation including both effective modes *x* and *y*. Dots: results from the inclusion of only the nonadiabatic coupling direction *y*, corresponding to asymmetric C–H stretching.

We note that the initially excited *B*_1*u*_(1sπ*) state undergoes rapid depopulation due to (i) strong coupling to the *B*_2*g*_(1sπ*) state, and; (ii) the near-zero energy difference between the two states. By 5.5 fs, 44% of the population has been transferred to the *B*_2*g*_(1sπ*) state, whereafter population flows back into the initially excited *B*_1*u*_(1sπ*) state. These dynamics occur within the Auger decay lifetime, which is estimated to be around 7 fs.^26^

To visualize the core-hole localization dynamics, we consider what we shall term the “core-hole asymmetry”, δ­(***R***, *t*), defined as the difference between the integral (over electronic coordinates) of the vibronic core-hole density projected onto the spaces spanned by the two different localized 1*s* orbitals:

$$\delta \left(R,t\right)=\int dr\left[{\mathrm{P̂}}_{1s_{r}}\left(r;R\right)\Delta \rho^{\left(core\right)}\left(r,R,t\right)-{\mathrm{P̂}}_{1s_{l}}\left(r;R\right)\Delta \rho^{\left(core\right)}\left(r,R,t\right)\right]$$ 12

where *P̂*_1*s*_*l*__(**r**; ***R***) and *P̂*_1*s*_*r*__(**r**; ***R***) denote the projectors onto the left/right carbon 1*s* AOs, which are localized on the left- and right-hand-side carbon atoms and denoted by 1*s*_*l*_ and 1*s*_*r*_. Notions of “left” and “right” here are, of course, ill-defined, but the meaning should still be clear. A negative value corresponds to a net accumulation of core-hole density on the left-hand-side carbon atom, and positive values to a net accumulation on the right-hand-side carbon atom. A value of zero corresponds to a completely delocalized vibronic core-hole density.

Figure 3 shows the core-hole asymmetry δ­(*y*, *t*) computed following vertical excitation to the *B*_1*u*_(1sπ*) state. Figure 3 shows the core-hole asymmetry δ­(***R***, *t*) computed following vertical excitation to the *B*_1*u*_(1sπ*) state. As discussed above, the core-hole dynamics in this case are governed almost entirely by the nonadiabatic coupling mode *y*, and only this nuclear degree of freedom was included in these calculations. The resulting computed core-hole asymmetry shall thus be denoted by δ­(*y*, *t*) in the following to emphasize this detail. At time *t* = 0, the core-hole asymmetry is zero for all geometries, corresponding to a vibronic core-hole density Δρ^(*core*)^(**r**, ***R***, *t*) is that completely delocalized. This in turn is a result of only the *B*_1*u*_(1sπ*) state being populated, and the coherence contribution Δρ_*coh*_^(*core*)^(**r**, ***R***, *t*) being zero. By around 2 fs, the core-hole becomes partially localized, as shown by the development of small, but nonzero core-hole asymmetry values at displaced values of the coupling mode *y*. This is caused by the transfer of population to the *B*_2*g*_(1sπ*) state and the coherence contribution Δρ_*coh*_^(*core*)^(**r**, ***R***, *t*) to the core-hole density beginning to grow. However, a significant nuclear probability density still exists at the *D*_2*h*_ ground state minimum energy geometry (*y* = 0), at which the core-hole remains delocalized. By 5.5 fs, when the difference in the *B*_1*u*_(1sπ*) and *B*_2*g*_(1sπ*) state populations approaches a minimum, and the coherence contribution reaches its maximum, the localization of the vibronic core-hole density reaches its maximum, as reflected in the large absolute values of δ­(*y*, *t*) at displaced values of the coupling mode *y*. These core-hole dynamics map directly onto the time-evolution of the nuclear wave function products χ_1_^*^(*y*, *t*)χ_2_(*y*, *t*), the absolute values of which are shown in Figure S4. We note that, due to symmetry, the value of the nuclear-coordinate-averaged electronic coherence, ⟨χ_1_|χ_2_⟩, is zero for all time.^27^ However, the spatially resolved electronic coherences χ_1_^*^(*y*, *t*)­χ_2_(*y*, *t*) at any particular value of *y* maybe nonzero.

**Figure 3:**
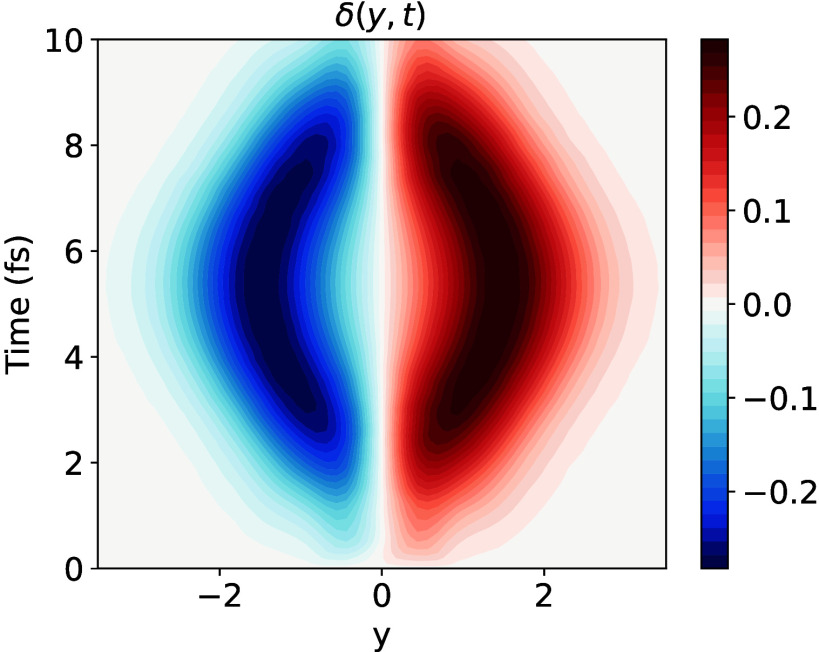
Core-hole asymmetry δ­(*y*, *t*) following excitation to the *B*_1*u*_(1sπ*) state. Negative values correspond to a net accumulation of core-hole density on the left-hand-side carbon atom, and positive values to a net accumulation on the right-hand-side carbon atom.

To quantify the degree of core-hole localization, we introduce the core-hole localization index *L*(*t*), defined as

L(t)=∫dR|δ(R,t)| 13

The localization index *L*(*t*) corresponds to the integral of the absolute value of the core-hole asymmetry, δ­(***R***, *t*), over nuclear coordinates. For singly core-excited states within the CVS approximation, this has limiting values of zero and one, corresponding to complete delocalization and localization of the core-hole density, respectively. The core-hole localization index following excitation to the *B*_1*u*_(1sπ*) state is shown in Figure 4. At time *t* = 0, the core-hole density is completely delocalized over the two carbon atoms, a result of only one of the two electronic states being populated and the population contribution, Δρ_*pop*_^(*core*)^(**r**, ***R***, *t*), dominating. Rapidly, however, core-level charge transfer occurs, causing the core-hole to localize, which is encoded in the increasing value of the core-hole localization index *L*(*t*). This increase tracks the population dynamics, and the value of *L*(*t*) reaches a maximum of 0.96 at 5.5 fs, corresponding to an almost entirely localized core hole. This is the time at which the population is most equally spread over the *B*_1*u*_(1sπ*) and *B*_2*g*_(1sπ*) states. Importantly, these dynamics occur within the estimated core-hole lifetime of around 7 fs.^26^ Therein after, the core hole begins to delocalize again as population flows back into the initially excited *B*_1*u*_(1sπ*) state.

**Figure 4:**
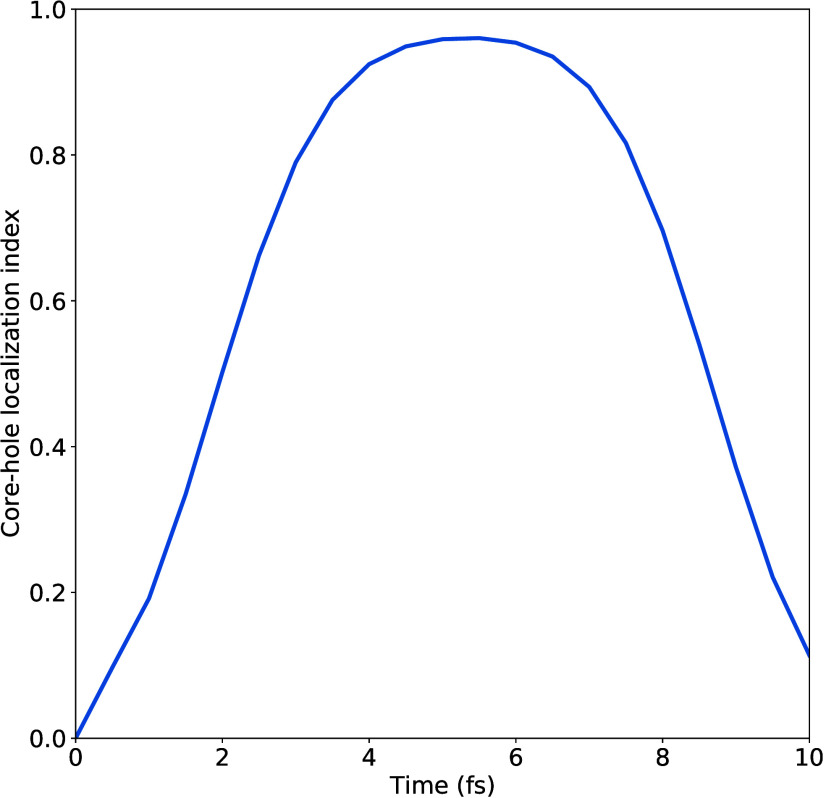
Core-hole localization index *L*(*t*) following excitation to the *B*_1*u*_(1sπ*) state. A value of zero corresponds to complete core-hole delocalization over the two carbon atoms and a value of one to complete core-hole localization on a single carbon atom.

To conclude, we have demonstrated that few-fs core-level charge transfer may occur following X-ray absorption, leading to ultrafast core-hole localization. Using the example of ethylene excited to its 1sπ* manifold by an ultrafast X-ray pump pulse, we predict that the initially delocalized core-hole will undergo near-total localization within around 5 fs, driven by core-level charge transfer. This is followed by core-hole delocalization, all of which occurs within the Auger decay window. The mechanism for these dynamics results from a breakdown of the Born–Oppenheimer approximation, and is driven by high-frequency asymmetric C–H stretching, corresponding to the Franck–Condon point nonadiabatic coupling direction for the two 1sπ* states involved. Importantly, core-level charge transfer in this case is predicted to occur on the same few-fs time scale as charge migration. It is interesting to note that, in both cases, the electronic dynamics are driven by the formation of coherences. In the cases of charge migration, these are created by the pump laser pulse, and nuclear dynamics can lead to their rapid destruction.^28^ In the case considered here, however, it is the nuclear motion that creates the coherences and drives the electronic dynamics. We anticipate that this phenomenon will be common to any molecule for which manifolds of nearly degenerate core-excited or core-ionised states exist, corresponding to the excitation of two or more equivalent 1*s* core orbitals which, for organic molecules with multiple equivalent atoms is the rule rather than the exception. Moreover, as the core-level charge transfer process occurs within the Auger decay window, it is in principle observable using modern X-ray light sources.^29,30^

## Supplementary Material



## Notes

Model Hamiltonian, details of the QD-DFT/MRCI(2) and quantum dynamics calculations, assessment of the validity of the effective mode and vertical excitation approximations, and details on the calculation of the one-electron vibronic reduced density and the core-hole localization index (PDF)
