# Supplementary material for: Femtosecond Core-Level Charge Transfer
Source: J Phys Chem Lett. 2025 Aug 21;16(35):8885–90. doi: 10.1021/acs.jpclett.5c01485 (PMC12415889; doi:10.1021/acs.jpclett.5c01485)
Supplement: Supplementary file 1 [file jz5c01485_si_001.pdf]

Supplementary Information for: Femtosecond Core-Level Charge Transfer

Simon P. Neville,[1,*] Martha Yaghoubi Jouybari,[2] and Michael Schuurman[1,3,†]

[1]*National Research Council Canada,*
100 Sussex Drive, Ottawa, Ontario K1A 0R6, Canada

[2]*Department of Chemistry and Biomolecular Sciences, Canada*

[3]*Department of Chemistry and Biomolecular Sciences,*
University of Ottawa, 10 Marie Curie,
Ottawa, Ontario, K1N 6N5, Canada

(Dated: July 21, 2025)

MODEL HAMILTONIAN

The starting point for our effective mode Hamiltonian is the construction of a full, 12-mode Hamiltonian expressed in terms of the ground state mass- and frequency-weighted normal modes Q_α. The Hamiltonian is represented in terms of a diabatic electronic state basis $\{|\psi_I(\boldsymbol{Q})\rangle\}$,

$$\begin{aligned}\hat{H} &= \sum_{I,J} |\psi_I(\boldsymbol{Q})\rangle\langle\psi_I(\boldsymbol{Q})|\hat{H}|\psi_J(\boldsymbol{Q})\rangle\langle\psi_J(\boldsymbol{Q})| \\ &= \sum_{I,J} |\psi_I(\boldsymbol{Q})\rangle\langle\psi_I(\boldsymbol{Q})|\hat{T} + \hat{H}_{el}|\psi_J(\boldsymbol{Q})\rangle\langle\psi_J(\boldsymbol{Q})| \\ &= \sum_{I,J} \delta_{IJ}\hat{T} + W_{IJ}(\boldsymbol{Q}).\end{aligned} \tag{1}$$

Here, $\hat{T}$ denotes the nuclear kinetic energy operator, which takes the form

$$\hat{T} = -\frac{1}{2}\sum_{\alpha}\omega_\alpha \frac{\partial^2}{\partial Q_\alpha^2}, \tag{2}$$

where ω_α is the frequency of the normal mode Q_α. $\hat{H}_{el}$ is the electronic Hamiltonian, and the $W_{IJ}(\boldsymbol{Q})$ are the elements of the diabatic potential matrix,

$$W_{IJ}(\boldsymbol{Q}) = \langle\psi_I(\boldsymbol{Q})|\hat{H}_{el}|\psi_J(\boldsymbol{Q})\rangle. \tag{3}$$

We work within the vibronic coupling Hamiltonian framework of Köppel, Domcke, and Cederbaum[1–3], and expand the diabatic potential matrix elements in a truncated Taylor series:

$$W_{IJ}(\boldsymbol{Q}) \approx \tau_0^{(I,J)} + \sum_{p=1}^{M}\frac{1}{p!}\sum_{\alpha}\tau_{p\alpha}^{(I,J)}Q_\alpha^p. \tag{4}$$

We take the expansion order, M, as 6. That is, we take a truncated 6th-order Taylor expansion of the diabatic potential, retaining only the one-mode terms. We fix the global gauge of the adiabatic-to-diabatic transformation by taking the adiabatic and diabatic representations to be equal at the Frank-Condon point, $\boldsymbol{Q}_0$. Then, the off-diagonal zeroth-order coefficients $\tau_0^{(I,J)}$, $I \neq J$, are zero by construction, whilst the on-diagonal coefficients $\tau_0^{(I,I)}$ are simply given by the adiabatic vertical excitation energies of the $B_{1u}(1s\pi^*)$ and $B_{2g}(1s\pi^*)$ state at $\boldsymbol{Q}_0$. The remaining expansion coefficients $\tau_{p\alpha}^{(I,J)}$ are given by

$$\tau_{p\alpha}^{(I,J)} = \left. \frac{\partial^p W_{IJ}}{\partial Q_\alpha^p} \right|_{\boldsymbol{Q}_0}, \tag{5}$$

and were determined by direct fitting to diabatic potential matrix elements computed using the QD-DFT/MRCI(2) method[4], using the normal equations approach detailed in Reference 5.

The effective modes x and y correspond, respectively, to the gradient difference and non-adiabatic coupling directions with respect to the $B_{1u}(1s\pi^*)$ and $B_{2g}(1s\pi^*)$ states. Let these correspond to the diabatic states $|\psi_1(\boldsymbol{Q})\rangle$ and $|\psi_2(\boldsymbol{Q})\rangle$, respectively. The effective modes are obtained from the full set of normal modes $\{Q_\alpha\}$ via the transformation

$$\begin{bmatrix} x \\ y \end{bmatrix} = \boldsymbol{U}^T \boldsymbol{Q}, \tag{6}$$

where the columns of $\boldsymbol{U}$ are given by

$$[\boldsymbol{U}]_{\alpha 1} = \frac{\frac{1}{2}\left(\tau_{1\alpha}^{(2,2)} - \tau_{1\alpha}^{(1,1)}\right)}{g}, \tag{7}$$

and

$$[\boldsymbol{U}]_{\alpha 2} = \frac{\tau_{1\alpha}^{(1,2)}}{h}, \tag{8}$$

where

$$g = \left|\left| \frac{1}{2}\left(\boldsymbol{\tau}_1^{(2)} - \boldsymbol{\tau}_1^{(1)}\right) \right|\right|, \tag{9}$$

and

$$h = \left|\left| \boldsymbol{\tau}_1^{(1,2)} \right|\right|. \tag{10}$$

In terms of the effective modes x and y, the kinetic energy operator and model diabatic potentials read as

$$\hat{T} = \frac{1}{2}\left(\tilde{\omega}_x \frac{\partial^2}{\partial x^2} + \tilde{\omega}_y \frac{\partial^2}{\partial y^2} \right) \tag{11}$$

and

$$W_{IJ}(x,y) \approx \tau_0^{(I,J)} + \sum_{p=1}^{M} \frac{1}{p!} \left(\tilde{\tau}_{px}^{(I,J)} x^p + \tilde{\tau}_{py}^{(I,J)} y^p \right), \tag{12}$$

respectively. Here, $\tilde{\omega}_x$ and $\tilde{\omega}_x$ are the frequencies of the effective modes, and are computed as

$$\begin{bmatrix} \tilde{\omega}_x & 0 \\ 0 & \tilde{\omega}_y \end{bmatrix} = \boldsymbol{U}^T \begin{bmatrix} \omega_x & 0 \\ 0 & \omega_y \end{bmatrix} \boldsymbol{U}, \tag{13}$$

where the diagonal form of the left-hand side of Equation 13 is a result of x and y having different (a_g and b_{3u}) symmetries. The transformed coupling coefficients $\tilde{\tau}_{px}^{(I,J)}$ and $\tilde{\tau}_{py}^{(I,J)}$ are computed as

$$\left(\tilde{\tau}_{px}^{(I,J)}, \tilde{\tau}_{py}^{(I,J)} \right)^T = \boldsymbol{U}^T \boldsymbol{\tau}_p^{(I,J)}. \tag{14}$$

QD-DFT/MRCI(2) CALCULATIONS

The diabatic potential matrix element values needed to fit the coupling coefficients $\tau_{p\alpha}^{(I,J)}$ were computed using the recently introduced QD-DFT/MRCI(2) method[4]. The QD-DFT/MRCI(2) approach exploits the intimate relationships between diabatisation, block diagonalisation of the potential matrix, and effective Hamiltonian theory to arrive at a procedure for the *direct* calculation of diabatic potentials and couplings. That is, it obviates the need to first compute the adiabatic potentials and then transform to a diabatic representation. Furthermore, the method is embedded within the powerful DFT/MRCI(2) framework[6, 7], which allows for the fast, accurate computation of excited electronic states in a completely black-box manner. In the QD-DFT/MRCI(2) calculations, the def2-TZVP basis was used. Two-electron integrals were evaluated using the density fitting approximation[8]. All calculations were performed using the General Reference Configuration Interaction (GRaCI) package[9], which is interfaced to the PySCF library[10] for the Kohn-Sham DFT and integral transformation components of the QD-DFT/MRCI(2) calculation. Core-excited states were computed via the application of the core-valence separation (CVS) approximation[11–13], using the CVS-QE12 DFT/MRCI Hamiltonian[14].

For reference, in Table S1 we give the values of the QD-DFT/MRCI(2)/def2-TZVP vertical excitation energies of the $B_{1u}(1s\pi^*)$ and $B_{2g}(1s\pi^*)$ states compared to benchmark-

State	QD-DFT/MRCI(2)	CCSDT
$B_{1u}(1s\pi^*)$	285.55	285.06
$B_{2g}(1s\pi^*)$	285.57	285.07

TABLE S1. Comparison of the Franck-Condon point vertical excitation energies of the $B_{1u}(1s\pi^*)$ and $B_{2g}(1s\pi^*)$ states computed at the QD-DFT/MRCI(2)/def2-TZVP and CCSDT/aug-cc-pCVTZ levels of theory. The QD-DFT/MRCI(2) values were computed using the CVS-QE12 Hamiltonian. All values are given in units of eV.

quality values computed at the CCSDT/aug-cc-pCVTZ level of theory. The error in the QD-DFT/MRCI(2) values is estimated to be around 0.5 eV. More importantly, however, the splitting of the $B_{1u}(1s\pi^*)$ and $B_{2g}(1s\pi^*)$ states furnished by the QD-DFT/MRCI(2) approach is found to be in excellent agreement with the CCSDT values, with values of 0.01 and 0.02 eV, respectively. We thus have some confidence in the performance of the QD-DFT/MRCI(2) method for the system considered.

EFFECTIVE MODE QUANTUM DYNAMICS SIMULATIONS

The nuclear wave functions $\chi_I(x, y, t)$, $I = 1, 2$, for the effective mode calculations were represented numerically exactly in terms of a direct product expansion in terms of harmonic oscillator discrete variable representation (DVR) basis sets[15] $\{\theta_i^{(x)}(x)\}$ and $\{\theta_i^{(y)}(y)\}$:

$$\chi_I(x, y, t) = \sum_{i_x=1}^{N_x} \sum_{i_y=1}^{N_y} C_{i_x,i_y}^{(I)}(t) \theta_{i_x}^{(x)}(x, t) \theta_{i_y}^{(y)}(y, t). \tag{15}$$

The time-evolution of the nuclear wave functions $\chi_I(x, y, t)$ was computed using the short iterative Lanczos algorithm[16] using an in-house developed code.

FULL-DIMENSIONAL QUANTUM DYNAMICS SIMULATIONS

Additional full-dimensional quantum dynamics simulations were performed using the multi-layer multiconfigurational time-dependent Hartree (ML-MCTDH) method[17–20]. The chosen ML-MCTDH tree corresponds to matrix product states (MPS) type *ansatz*, which may be expressed as follows. First, we start with the numerically exact direct prod-

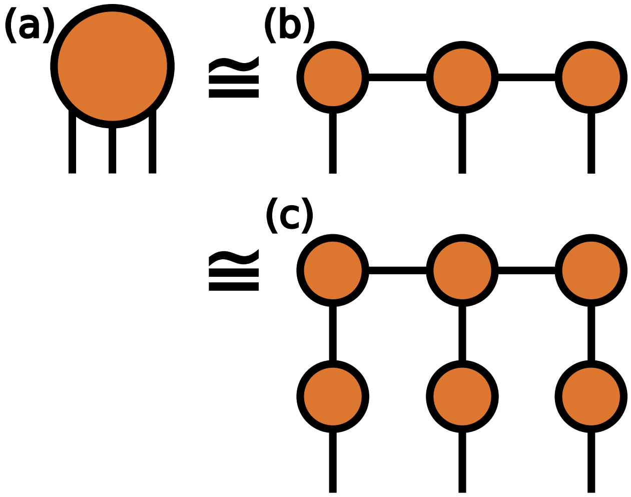

FIG. S1. Diagrammatic representation of the wave function *ansatz* for three physical degrees of freedom. (a) Numerically exact coefficient tensor $C^{(I)}(t) \in \mathbb{C}^{N_1 \times \cdots \times N_d}$, (b) MPS approximation of the coefficient tensor in terms of products of tensors $A^{(k;I)} \in \mathbb{C}^{r_{k-1} \times N_k \times r_k}$, (c) further expansion of each tensor $A^{(k;I)}$ in terms of tensors $b^{(k;I)}(t) \in \mathbb{C}^{r_{k-1} \times s_k \times r_k}$ and $c^{(k;I)}(t) \in \mathbb{C}^{s_k \times N_k}$.

uct expansion of each d-dimensional nuclear wave function $\chi_I(Q_1, Q_2, \ldots, Q_d, t)$, $I = 1, 2$ in terms of DVR basis functions $\{\theta_i^{(k)}(Q_k)\}$:

$$\chi_I(Q_1, Q_2, \ldots, Q_d, t) = \sum_{i_1=1}^{N_1} \sum_{i_2=1}^{N_2} \cdots \sum_{i_d=1}^{N_d} C^{(I)}_{i_1, i_2, \ldots, i_d}(t) \prod_{k=1}^{d} \theta_{i_k}^{(k)}(Q_k, d). \tag{16}$$

The coefficient tensor $C^{(I)}(t)$ is then approximated using the following MPS expansion:

$$C^{(I)}_{i_1, i_2, \ldots, i_d}(t) \approx \sum_{\alpha_0=1}^{r_0} \sum_{\alpha_1=1}^{r_1} \cdots \sum_{\alpha_d}^{r_d} A^{(1;I)}_{i_1, \alpha_1}(t) A^{(2;I)}_{\alpha_1, i_2, \alpha_2}(t) \cdots A^{(d;I)}_{\alpha_{d-1}, i_d}(t), \tag{17}$$

with $\alpha_0 = \alpha_d = 1$. Finally, each tensor $A^{(k;I)}$ is further expanded as

$$A^{(k;I)}_{\alpha_{k-1}, i_k, \alpha_k}(t) = \sum_{\beta=1}^{s_k} b^{(k;I)}_{\alpha_{k\_1}, \beta, \alpha_k}(t) c^{(k;I)}_{\beta, i_k}(t). \tag{18}$$

The diagrammatic form of this wave function *ansatz* is given in Figure S1 for the case of three vibrational degrees of freedom ($d = 3$). Here, each node represents a tensor, and each vertex a tensor dimension. Vertices connecting neighbouring tensors correspond to a contraction over a shared index. The remaining unconnected vertices correspond to physical degrees of freedom (that is, modes). Figure S1(a) shows the full-format coefficient

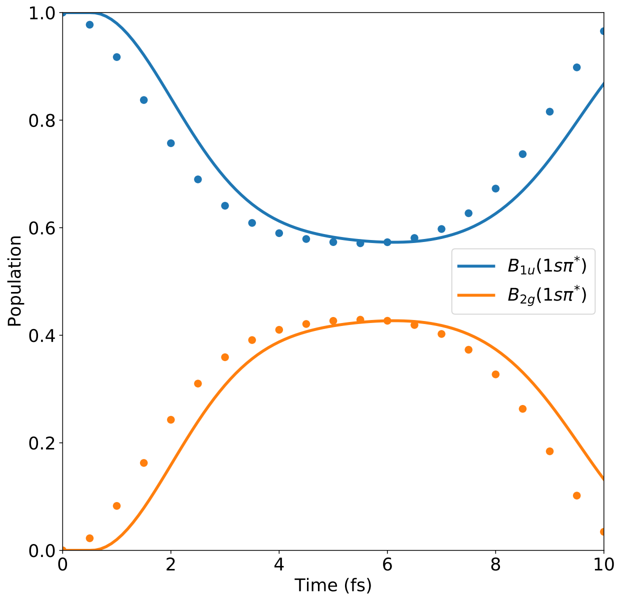

FIG. S2. Diabatic $B_{1u}(1s\pi^*)$ and $B_{2g}(1s\pi^*)$ state populations following vertical excitation to the $B_{1u}(1s\pi^*)$ state. Dots: effective two-mode model. Solid lines: full dimensional 12-mode model.

tensor $C^{(I)}(t) \in \mathbb{C}^{N_1\times\cdots\times N_d}$ for the case of three modes ($d = 3$). Figure S1(b) shows the corresponding MPS approximation of the coefficient tensor, and, finally, the wave function *ansatz* used in this work is given in Figure S1(c). This corresponds to a special case of an ML-MCTDH wave function, with d layers and $2d$ nodes. As such, the powerful machinery built around this method may be leveraged. The parameters of the wave function are propagated variationally according to the Dirac-Frenkel variational principle. For all wave packet propagations, the Heidelberg MCTDH package was used[21].

VALIDITY OF THE EFFECTIVE MODE TREATMENT

In order to assess the validity of the effective mode model, we consider the population dynamics following vertical excitation to the $B_{1u}(1s\pi^*)$ state using both the full 12-mode and reduced 2-effective-mode Hamiltonians. A comparison of these results are shown in Figure S2. Within the short timescales considered, the two models agree very well, with both the timescales for internal conversion to the $B_{2g}(1s\pi^*)$ state as well as the amount of population transferred being very similar. We thus consider the effective mode model to capable of correctly describing the short (<10 fs) dynamics following excitation to the

$B_{1u}(1s\pi^*)$ state.

VALIDITY OF THE VERTICAL EXCITATION APPROXIMATION

We are interested in the broadband X-ray excitation of ethylene to its $1s\pi^*$ manifold, spanned by the $B_{1u}(1s\pi^*)$ and $B_{2g}(1s\pi^*)$ states, and corresponding to the first absorption band at the C K-edge. In the main text, we have assumed that if a sufficiently short pump laser pulse is used, then the initial wave packet prepared should reasonably be described by vertical excitation to the bright $B_{1u}(1s\pi^*)$. To demonstrate the validity of this approximation, further quantum dynamics simulations were performed in which the interaction of the ground vibronic state with a 2 fs X-ray pump pulse was included. The molecular Hamiltonian was modified to contain a third electronic state corresponding to the ground state within the harmonic approximation. The interaction with the pump laser pulse was included within dipole approximation via the addition of the light-matter interaction operator $\hat{H}_{ML}(t)$ to the molecular Hamiltonian $\hat{H}$:

$$\hat{H} \rightarrow \hat{H} + \hat{H}_{ML}(t), \tag{19}$$

$$\begin{aligned} \hat{H}_{ML}(t) &= -\sum_{I,J} |\psi_I(\boldsymbol{Q})\rangle \langle \psi_I(\boldsymbol{Q})| \hat{\boldsymbol{\mu}} \cdot \boldsymbol{\epsilon}(t) |\psi_J(\boldsymbol{Q})\rangle \langle \psi_J(\boldsymbol{Q})| \\ &= -\sum_{IJ} |\psi_I(\boldsymbol{Q})\rangle \boldsymbol{M}_{IJ}(\boldsymbol{Q}) \langle \psi_J(\boldsymbol{Q})|. \end{aligned} \tag{20}$$

Here, $\boldsymbol{\epsilon}(t)$ denotes the external electric field, and $\boldsymbol{M}(\boldsymbol{Q})$ is the diabatic state representation of the dipole operator,

$$M_{IJ}(\boldsymbol{Q}) = \langle \psi_I(\boldsymbol{Q})|\hat{\boldsymbol{\mu}}|\psi_J(\boldsymbol{Q})\rangle. \tag{21}$$

We work within the Condon approximation, and assume that the diabatic dipole matrix $\boldsymbol{M}(\boldsymbol{Q})$ is nuclear-coordinate-independent. By making use of the equivalence of the adiabatic and diabatic representations at the Franck-Condon point $\boldsymbol{Q}_0$ within our model, the elements of $\boldsymbol{M}(\boldsymbol{Q})$ may then be simply equated with values of the adiabatic dipoles and transition dipoles computed at the Frank-Condon point. The external electric field $\boldsymbol{\epsilon}(t)$ is modeled using a normalized Gaussian envelope,

$$\boldsymbol{\epsilon}(t) = \boldsymbol{e}\left(\frac{S}{\sigma}\right)\sqrt{\frac{4\ln 2}{\pi}}\exp\left[-\frac{4\ln 2}{\sigma^2}\left(t-t_0\right)\right]\cos\left[\omega\left(t-t_0\right)\right], \tag{22}$$

where $\boldsymbol{e}$ represents the polarization vector, σ is the full width at half maximum (FWHM) of the pulse, ω is the central frequency, t_0 is the centre of the pulse, and S is a strength parameter used to control the peak intensity. Parameter values of σ=2.0 fs, ω=285.3 eV, t_0=0.0 fs, and S=3.0 au were used. The central frequency used corresponds to the centre of the first C K-edge absorption band within the model, and the FWHM was chosen such that the interaction with the pump laser pulse occurs well within the Auger decay window.

Shown in Figure S3 are the diabatic $B_{1u}(1s\pi^*)$ state populations resulting from the interaction of the ground vibronic state with the pump X-ray laser pulse. The dynamics are found to be essentially the same as for vertical excitation to the $B_{1u}(1s\pi^*)$ state. Importantly, even though the $B_{1u}(1s\pi^*)$ and $B_{2g}(1s\pi^*)$ states are strongly vibronically coupled, only the $B_{1u}(1s\pi^*)$ is directly populated by the pump pulse, as evidenced by the delayed rise of the $B_{2g}(1s\pi^*)$ state population relative to that of the $B_{1u}(1s\pi^*)$ state. Additionally, the population dynamics after time $t = 0$ are very similar to those following vertical excitation to the $B_{1u}(1s\pi^*)$, both in terms of the timescale for internal conversion to the $B_{2g}(1s\pi^*)$ state and the proportion of population transferred. As such, the approximation of such a few-fs, broadband X-ray pump process by vertical excitation to the $B_{1u}(1s\pi^*)$ state appears to be justified.

CALCULATION OF ONE-ELECTRON VIBRONIC REDUCED DENSITY

The central ingredient in the calculation and analysis of the vibronic core-hole density is the one-electron vibronic reduced density matrix (1-VRD), $\rho(\mathbf{r};\boldsymbol{R},t)$. We here detail the procedure for its contruction using the combined results of quantum dynamics and quantum chemistry calculations. First, for practical purposes, the position-dependent molecular orbital (MO) representation of $\rho(\mathbf{r},\boldsymbol{R},t)$ needs to be introduced. Let $\{\varphi_p(\mathbf{r};\boldsymbol{R})\}$ denote the chosen set of MOs used to represent the electronic wave functions at nuclear geometry $\boldsymbol{R}$. Then, the vibronic one-electron reduced density may be expressed as

$$\rho(\mathbf{r},\boldsymbol{R},t) = \sum_{p,q} D_{pq}(\boldsymbol{R},t)\varphi_p^*(\mathbf{r};\boldsymbol{R})\varphi_q(\mathbf{r};\boldsymbol{R}), \tag{23}$$

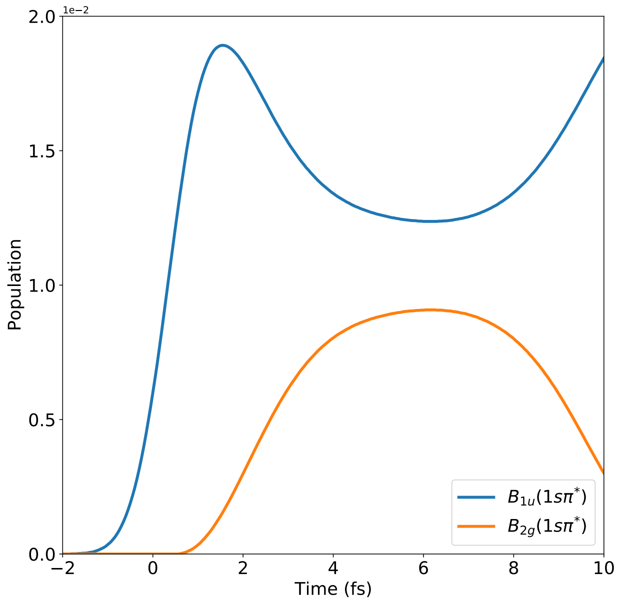

FIG. S3. Diabatic $B_{1u}(1s\pi^*)$ and $B_{2g}(1s\pi^*)$ state populations resulting from the interaction of the ground state with a 2 fs X-ray pump pulse with a central frequency of 285.3 eV, corresponding to the centre of the first band in the C K-edge absorption spectrum

where $\boldsymbol{D}(\boldsymbol{R}, t)$ is what we shall term the "one-electron *vibronic* reduced density matrix", or 1-VRDM, in order to distinguish it from the usual one-electron reduced density matrix (1-RDM) encountered in quantum chemistry. The 1-VDM is the MO representation of the density $\rho(\mathbf{r}, \boldsymbol{R}, t)$, with elements given by

$$D_{pq}(\boldsymbol{R}, t) = \left\langle \Psi(\boldsymbol{r}, \boldsymbol{R}, t) \middle| \hat{a}_p^\dagger(\boldsymbol{R}) \hat{a}_q(\boldsymbol{R}) \middle| \Psi(\boldsymbol{r}, \boldsymbol{R}, t) \right\rangle, \tag{24}$$

where $\hat{a}_p^\dagger(\boldsymbol{R})$ ($\hat{a}_p(\boldsymbol{R})$) is the elementary Fermionic creation (annihilation) operator associated with MO $\varphi_p(\mathbf{r}; \boldsymbol{R})$.

Inserting the Born-Huang expansion of $\Psi(\boldsymbol{r}, \boldsymbol{R}, t)$ into Equation 24 allows us to express the 1-VRDM in terms of population and coherence contributions,

$$\boldsymbol{D}(\boldsymbol{R}, t) = \boldsymbol{D}^{(pop)}(\boldsymbol{R}, t) + \boldsymbol{D}^{(coh)}(\boldsymbol{R}, t), \tag{25}$$

$$\boldsymbol{D}^{(pop)}(\boldsymbol{R}, t) = \sum_I = \left|\chi_I(\boldsymbol{R}, t)\right|^2 \boldsymbol{D}^{(I,I)}(\boldsymbol{R}), \tag{26}$$

$$\boldsymbol{D}^{(coh)}(\boldsymbol{R},t) = \sum_{I \neq J} = \chi_I^*(\boldsymbol{R},t)\chi_J(\boldsymbol{R},t)\boldsymbol{D}^{(I,J)}(\boldsymbol{R}), \tag{27}$$

where the $\boldsymbol{D}^{(I,J)}(\boldsymbol{R})$ are the usual one-electron reduced (transition) density matrices (1-RDMs [1-TDMs]):

$$D_{pq}^{(I,J)}(\boldsymbol{R}) = \left\langle \psi_I(\boldsymbol{r};\boldsymbol{R}) \middle| \hat{a}_p^\dagger(\boldsymbol{R})\hat{a}_q(\boldsymbol{R}) \middle| \psi_j(\boldsymbol{r};\boldsymbol{R}) \right\rangle. \tag{28}$$

The $\boldsymbol{D}^{(I,J)}(\boldsymbol{R})$ are computed in the diabatic representation using the QD-DFT/MRCI(2) wave functions, while the nuclear wave functions $\chi_I(\boldsymbol{R},t)$ are extracted from the quantum dynamics simulations. Importantly, as the diabatic potential matrix used in the quantum dynamics simulations are based on the QD-DFT/MRCI(2) diabatic potentials, this results in the required consistency between the diabatic 1-RDMs (1-TDMs) and nuclear wave functions.

CALCULATION OF THE CORE-HOLE LOCALISATION INDEX

Working within the effective mode model, to compute the core-hole localisation index $L(t)$, the integral

$$L(t) = \int dxdy \; \mathrm{abs}\left[\delta(x,y,t)\right] \tag{29}$$

has to be evaluated. The core-hole asymmetry $\delta(x,y,t)$ is readily evaluated given the vibronic one-electron reduced density $\rho(\mathbf{r},x,y,t)$, and all that is left is to determine a suitable quadrature scheme for the evaluation of the integral over nuclear coordinates in Equation 29. For this, we note that the quantum dynamics simulations are performed using a harmonic oscillator DVR basis. This is a Gaussian quadrature DVR[15]. That is, the DVR grid points and weights, $\{(x_{i_x},y_{i_y})\}$ and $\{(w_{i_x}^{(x)},w_{i_y}^{(y)})\}$, respectively, correspond to Gaussian quadrature points and weights. This gives a simple, yet accurate, prescription for the calculation of the core-hole localisation index. Namely, the nuclear wave functions $\chi_I(x,y,t)$ are evaluated at the DVR grid points, and used to construct the vibronic one-electron reduced density matrix on the DVR grid, $\rho(\mathbf{r},x_{i_x},y_{i_y},t)$, from which the core-hole asymmetry on the DVR grid, $\delta(x_{i_x},y_{i_y},t)$, may be computed. Then, the core-hole localisation index may be calculated as

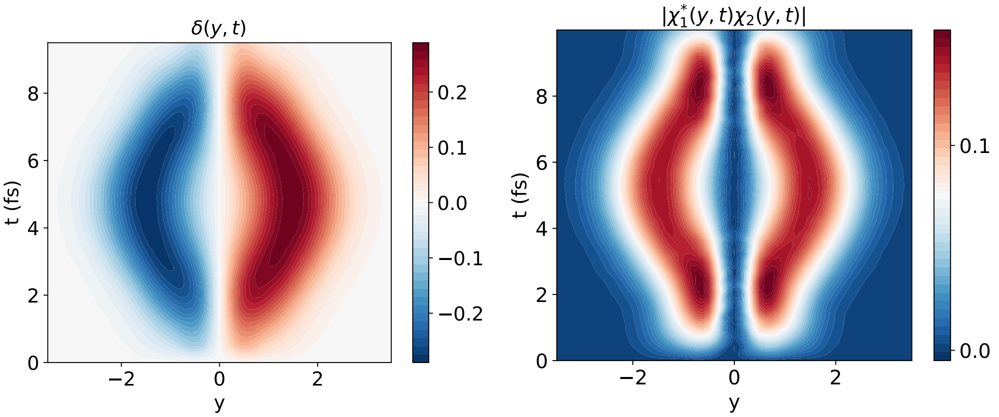

FIG. S4. Left: the core-hole asymmetry $\delta(y,t)$ following excitation to the $B_{1u}(1s\pi^*)$ state. Negative values correspond to a net accumulation of core-hole density on the left-hand-side carbon atom, and positive values to a net accumulation on the right-hand-side carbon atom. Right: absolute value of the product of the nuclear wave functions for the $B_{1u}(1s\pi^*)$ and $B_{2g}(1s\pi^*)$ states.

$$L(t) = \sum_{i_x=1}^{N_x} \sum_{i_y=1}^{N_y} \delta(x_{i_x}, y_{i_y}, t) w_{i_x}^{(x)} w_{i_y}^{(y)}. \tag{30}$$

NUCLEAR WAVE FUNCTION PRODUCTS $\chi_1^*(y,t)\chi_2(y,t)$

In Figure S4, we show the time-evolution of the nuclear wave function products $\chi_1^*(y,t)\chi_2(y,t)$ following excitation to the $B_{1u}(1s\pi^*)$ state. Reproduced from the main text, we show alongside the time-evolution of the core-hole asymmetry $\delta(y,t)$. It is clearly seen that the core-hole asymmetry, and indeed the core-hole dynamics themselves, directly follow the time-evolution of the product $\chi_1^*(y,t)\chi_2(y,t)$.

* Simon.Neville@nrc-cnrc.gc.ca

† Michael.Schuurman@uottawa.ca

[1] H. Köppel, W. Domcke, and L. S. Cederbaum, Multimode molecular dynamics beyond the born-oppenheimer approximation, in *Advances in Chemical Physics* (John Wiley Sons, Ltd, 1984) pp. 59–246.

[2] H. Köppel, W. Domcke, and L. S. Cederbaum, The multi-mode vibronic-coupling approach, in *Conical Intersections*, pp. 323–367, https://www.worldscientific.com/doi/pdf/10.1142/9789812565464$_0$007.*L. Cederbaum, W. Domcke, H. Köppe* [illegible] *The "mysteryband" ofbutatriene, ChemicalPhysics* **26**, 169(1977).

[4] S. P. Neville and M. S. Schuurman, Calculation of quasi-diabatic states within the DFT/MRCI(2) framework: The QD-DFT/MRCI(2) method, The Journal of Chemical Physics **160**, 234109 (2024), https://pubs.aip.org/aip/jcp/article-pdf/doi/10.1063/5.0214637/20004718/234109_1_5.0214637.pdf.

[5] S. P. Neville, I. Seidu, and M. S. Schuurman, Propagative block diagonalization diabatization of DFT/MRCI electronic states, The Journal of Chemical Physics **152**, 114110 (2020), https://pubs.aip.org/aip/jcp/article-pdf/doi/10.1063/1.5143126/15573990/114110_1_online.pdf.

[6] S. Grimme and M. Waletzke, A combination of kohn–sham density functional theory and multi-reference configuration interaction methods, The Journal of chemical physics **111**, 5645 (1999).

[7] S. P. Neville and M. S. Schuurman, A perturbative approximation to DFT/MRCI: DFT/MRCI(2), The Journal of Chemical Physics **157**, 164103 (2022), https://pubs.aip.org/aip/jcp/article-pdf/doi/10.1063/5.0118285/16551884/164103_1_online.pdf.

[8] B. I. Dunlap, Robust and variational fitting, Phys. Chem. Chem. Phys. **2**, 2113 (2000).

[9] S. Neville and M. Schuurman, GRaCI: General Reference Configuration Interaction (2021).

[10] Q. Sun, X. Zhang, S. Banerjee, P. Bao, M. Barbry, N. S. Blunt, N. A. Bogdanov, G. H. Booth, J. Chen, Z.-H. Cui, J. J. Eriksen, Y. Gao, S. Guo, J. Hermann, M. R. Hermes, K. Koh, P. Koval, S. Lehtola, Z. Li, J. Liu, N. Mardirossian, J. D. McClain, M. Motta, B. Mussard, H. Q. Pham, A. Pulkin, W. Purwanto, P. J. Robinson, E. Ronca, E. R. Sayfutyarova, M. Scheurer, H. F. Schurkus, J. E. T. Smith, C. Sun, S.-N. Sun, S. Upadhyay, L. K. Wagner, X. Wang, A. White, J. D. Whitfield, M. J. Williamson, S. Wouters, J. Yang, J. M. Yu, T. Zhu, T. C. Berkelbach, S. Sharma, A. Y. Sokolov, and G. K.-L. Chan, Recent developments in the PySCF program package, The Journal of Chemical Physics **153**, 024109 (2020), https://pubs.aip.org/aip/jcp/article-pdf/doi/10.1063/5.0006074/16722275/024109_1_online.pdf.

[11] L. S. Cederbaum, W. Domcke, and J. Schirmer, Many-body theory of core holes, Phys. Rev. A **22**, 206 (1980).

[12] A. Barth and L. S. Cederbaum, Many-body theory of core-valence excitations, Phys. Rev. A **23**, 1038 (1981).

[13] I. Seidu, S. P. Neville, M. Kleinschmidt, A. Heil, C. M. Marian, and M. S. Schuurman, The simulation of X-ray absorption spectra from ground and excited electronic states using core-valence separated DFT/MRCI, The Journal of Chemical Physics **151**, 144104 (2019), https://pubs.aip.org/aip/jcp/article-pdf/doi/10.1063/1.5110418/15565425/144104_1_online.pdf.

[14] T. S. Costain, J. B. Rolston, S. P. Neville, and M. S. Schuurman, A dft/mrci hamiltonian parameterized using only ab initio data. ii. core-excited states, The Journal of Chemical Physics **161**, 114117 (2024), https://pubs.aip.org/aip/jcp/article-pdf/doi/10.1063/5.0227385/20166310/114117_1_5.0227385.pdf.

[15] J. C. Light and T. Carrington Jr., Discrete-variable representations and their utilization, in *Advances in Chemical Physics* (John Wiley Sons, Ltd, 2000) pp. 263–310, https://onlinelibrary.wiley.com/doi/pdf/10.1002/9780470141731.ch4.

[16] T. J. Park and J. C. Light, Unitary quantum time evolution by iterative Lanczos reduction, The Journal of Chemical Physics **85**, 5870 (1986), https://pubs.aip.org/aip/jcp/article-pdf/85/10/5870/18962048/5870_1_online.pdf.

[17] H. Wang and M. Thoss, Multilayer formulation of the multiconfiguration time-dependent Hartree theory, The Journal of Chemical Physics **119**, 1289 (2003), https://pubs.aip.org/aip/jcp/article-pdf/119/3/1289/10852931/1289_1_online.pdf.

[18] U. Manthe, A multilayer multiconfigurational time-dependent Hartree approach for quantum dynamics on general potential energy surfaces, The Journal of Chemical Physics **128**, 164116 (2008), https://pubs.aip.org/aip/jcp/article-pdf/doi/10.1063/1.2902982/15410377/164116_1_online.pdf.

[19] U. Manthe, Layered discrete variable representations and their application within the multiconfigurational time-dependent Hartree approach, The Journal of Chemical Physics **130**, 054109 (2009), https://pubs.aip.org/aip/jcp/article-pdf/doi/10.1063/1.3069655/15422973/054109_1_online.pdf.

[20] O. Vendrell and H.-D. Meyer, Multilayer multiconfiguration time-dependent Hartree method: Implementation and applications to a Henon–Heiles Hamiltonian and to pyrazine, The Journal of Chemical Physics **134**, 044135 (2011), https://pubs.aip.org/aip/jcp/article-pdf/doi/10.1063/1.3535541/13789371/044135_1_online.pdf.

[21] G. A. Worth, M. H. Beck, A. Jäckle, and H.-D. Meyer, The MCTDH Package, Version 8.6. See http://mctdh.uni-hd.de.
